# Machine learning-based segmentation of ischemic penumbra by using diffusion tensor metrics in a rat model

**DOI:** 10.1186/s12929-020-00672-9

**Published:** 2020-07-15

**Authors:** Duen-Pang Kuo, Po-Chih Kuo, Yung-Chieh Chen, Yu-Chieh Jill Kao, Ching-Yen Lee, Hsiao-Wen Chung, Cheng-Yu Chen

**Affiliations:** 1 Department of Medical Imaging, Taipei Medical University Hospital, No.250, Wu-Hsing St, Taipei, 11031 Taiwan; 2 Department of Radiology, Taoyuan Armed Forces General Hospital, Taoyuan, Taiwan; 3 Institute for Medical Engineering and Science, Massachusetts Institute of Technology, Cambridge, MA USA; 4 Department of Biomedical Imaging and Radiological Sciences, National Yang-Ming University, No.155, Sec.2, Linong St, Taipei, 11221 Taiwan; 5 Department of Radiology, School of Medicine, College of Medicine, Taipei Medical University, No.250, Wu-Hsing St, Taipei, 11031 Taiwan; 6 TMU Center for Big Data and Artificial Intelligence in Medical Imaging, Taipei Medical University Hospital, Taipei, Taiwan; 7 TMU Research Center for Artificial Intelligence in Medicine, Taipei Medical University Hospital, Taipei, Taiwan; 8 Graduate Institute of Biomedical Electrics and Bioinformatics, National Taiwan University, Taipei, Taiwan; 9 Radiogenomic Research Center, Taipei Medical University Hospital, No.250, Wu-Hsing St, Taipei, 11031 Taiwan; 10 Center for Artificial Intelligence in Medicine, Taipei Medical University, No.250, Wu-Hsing St, Taipei, 11031 Taiwan; 11 Department of Radiology, National Defense Medical Center, No.250, Wu-Hsing St, Taipei, 11031 Taiwan

**Keywords:** Machine learning, Diffusion tensor imaging, Ischemic penumbra, Infarct core

## Abstract

**Background:**

Recent trials have shown promise in intra-arterial thrombectomy after the first 6–24 h of stroke onset. Quick and precise identification of the salvageable tissue is essential for successful stroke management. In this study, we examined the feasibility of machine learning (ML) approaches for differentiating the ischemic penumbra (IP) from the infarct core (IC) by using diffusion tensor imaging (DTI)-derived metrics.

**Methods:**

Fourteen male rats subjected to permanent middle cerebral artery occlusion (pMCAO) were included in this study. Using a 7 T magnetic resonance imaging, DTI metrics such as fractional anisotropy, pure anisotropy, diffusion magnitude, mean diffusivity (MD), axial diffusivity, and radial diffusivity were derived. The MD and relative cerebral blood flow maps were coregistered to define the IP and IC at 0.5 h after pMCAO. A 2-level classifier was proposed based on DTI-derived metrics to classify stroke hemispheres into the IP, IC, and normal tissue (NT). The classification performance was evaluated using leave-one-out cross validation.

**Results:**

The IC and non-IC can be accurately segmented by the proposed 2-level classifier with an area under the receiver operating characteristic curve (AUC) between 0.99 and 1.00, and with accuracies between 96.3 and 96.7%. For the training dataset, the non-IC can be further classified into the IP and NT with an AUC between 0.96 and 0.98, and with accuracies between 95.0 and 95.9%. For the testing dataset, the classification accuracy for IC and non-IC was 96.0 ± 2.3% whereas for IP and NT, it was 80.1 ± 8.0%. Overall, we achieved the accuracy of 88.1 ± 6.7% for classifying three tissue subtypes (IP, IC, and NT) in the stroke hemisphere and the estimated lesion volumes were not significantly different from those of the ground truth *(p* = .56, .94, and .78, respectively).

**Conclusions:**

Our method achieved comparable results to the conventional approach using perfusion–diffusion mismatch. We suggest that a single DTI sequence along with ML algorithms is capable of dichotomizing ischemic tissue into the IC and IP.

## Background

Stroke is one of the major causes of long-term disability and death, and nearly 80% of stroke cases are ischemic [1]. Treatment options for acute ischemic stroke (AIS) are rapid recanalization of the occluded large vessels by using intravenous (IV) thrombolysis with tissue plasminogen activator (tPA) and intra-arterial (IA) thrombectomy to mechanically disrupt or remove the thrombus. In either treatment, identifying a substantial and salvageable ischemic penumbra (IP) is essential for a patient to be eligible for therapy [2–4]. In the DAWN and DEFUSE 3 trials, which included acute stroke patients within 6–24 h of onset, obtaining perfusion imaging computed tomography (CT) perfusion or magnetic resonance imaging (MRI) perfusion-weighted imaging (PWI) or an MRI with a diffusion-weighted imaging (DWI) sequence was recommended to help determine whether the patient is a candidate for mechanical thrombectomy [2, 5]. In the acute setting, an infarct core (IC) can be identified through DWI and combined with the hypoperfusion area depicted by PWI, which allows for the specific definition of the salvageable IP and IC by using the concept of perfusion–diffusion mismatch (PDM). However, quick and accurate delineation of IP is demanded by clinicians for AIS management.

Diffusion tensor imaging (DTI) has been used in clinical applications for measuring cerebral microstructural changes induced by neurological diseases [6]. In AIS, DTI-derived metrics, such as fractional anisotropy (FA), mean diffusivity (MD), pure anisotropic diffusion (q), and diffusion magnitude (L), have demonstrated the feasibility of DTI in assessing the damage of ischemic brain tissue [7], determining the onset time of AIS in hours in an animal model [8] or in humans [9, 10], estimating the salvageable tissue [8], and microstructurally discriminating benign oligemia from the “true” penumbral tissue [11]. Based on the previous studies, DTI may provide comprehensive characterization of the pathophysiological process of cerebral ischemia.

Machine learning (ML) has become a useful aid for physicians in the diagnosis of, treatment of, and prediction of complications and patient outcomes for numerous diseases. While extracting meaningful and discriminative imaging features that exhibits the characteristics of lesion part [12, 13], ML-based algorithms can establish predictive models for various clinical applications [12, 14]. In the current study, we attempted to develop a 2-level ML classifier based on DTI-derived metrics for characterizing ischemic tissue subtypes. We aimed to determine whether the DTI sequence along with ML algorithms could classify the stroke hemisphere into the IP, IC, and unaffected normal tissue (NT) during the AIS stage.

## Materials and methods

### Animals

Fourteen male Sprague-Dawley rats (weight, 270–350 g; Taipei Medical University Animal Center, Taiwan) were used in this study. The rats were housed in a humidity- and temperature-controlled environment and placed under a 12:12-h light–dark cycle, with free access to sterile food and water. All the rats underwent permanent middle cerebral artery occlusion (pMCAO) through an intraluminal suture method based on the modified Zea Longa approach [15]. All animal experiments were approved and performed in accordance with guidelines and regulations of the Institutional Animal Care and Use Committee of Taipei Medical University (IACUC approval No: LAC-2015-0033).

### Image acquisition

Images were acquired using a 7 T MRI scanner (PharmaScan 70/16; Bruker Biospin, Ettlingen, Germany). During image acquisition, the rats were placed under anesthesia by using 1.5–2% isoflurane and the rectal temperature was maintained at approximately 37 °C using a warm water bath with continuous circulation through a water-bath temperature controller set outside the magnet. DTI was performed with 6 noncollinear diffusion encoding gradients with a b factor of 1200 s/mm^2^ and 1 b = 0 s/mm^2^. Multishot echoplanar imaging (repetition time [TR] = 3000 ms, echo time [TE] = 37 ms, number of excitations = 6) with the navigator-echo correction technique was used as the signal readout module. To obtain the largest IP area and avoid fast diminishing, PWI was performed once at 0.5 h after pMCAO by using a dynamic susceptibility contrast technique. A series of gradient-echo echoplanar coronal images with a TR/TE value of 1000/20 ms and 300 repetitions were acquired. A bolus of the susceptibility contrast agent gadolinium-diethylenetriamine penta-acetic acid (0.25 mmol/kg; Magnevist, Bayer Schering Pharma, Berlin, Germany) was injected manually through the rat tail vein approximately 30 s after the start of image acquisition. All the images acquired from DTI and PWI were with a field-of-view of 20 mm × 20 mm and a matrix of 64 × 64, which were subsequently zero-filled to 128 × 128 with a resolution of 0.16 mm × 0.16 mm for further analyses.

### Data analysis

#### Calculation of the relative cerebral blood flow and DTI metrics

The relative cerebral blood flow (rCBF) and DTI metrics were calculated using in-house algorithms in MATLAB (MathWorks, Natick, MA, USA). First, the relative cerebral blood volume (rCBV) and relative mean transit time (rMTT) were determined using the integral and normalized first moment of gamma variate fitting, respectively. Next, rCBF was derived as the quotient of rCBV divided by rMTT by using the central volume principle [16]. For DTI metrics, the eigenvalues of each image voxel were computed and then applied to derive the MD, FA, q, L, axial diffusivity (AD), and radial diffusivity (RD). To avoid noise or artifacts, all the DTI-derived and rCBF maps were computed through a convolution with a Gaussian kernel using the weighted mean intensity value. All the smoothed maps were then normalized linearly to the range [0, 1] for inter-rat comparisons.

#### Delineation of the IP, IC, and NT

**Fig. 1:**
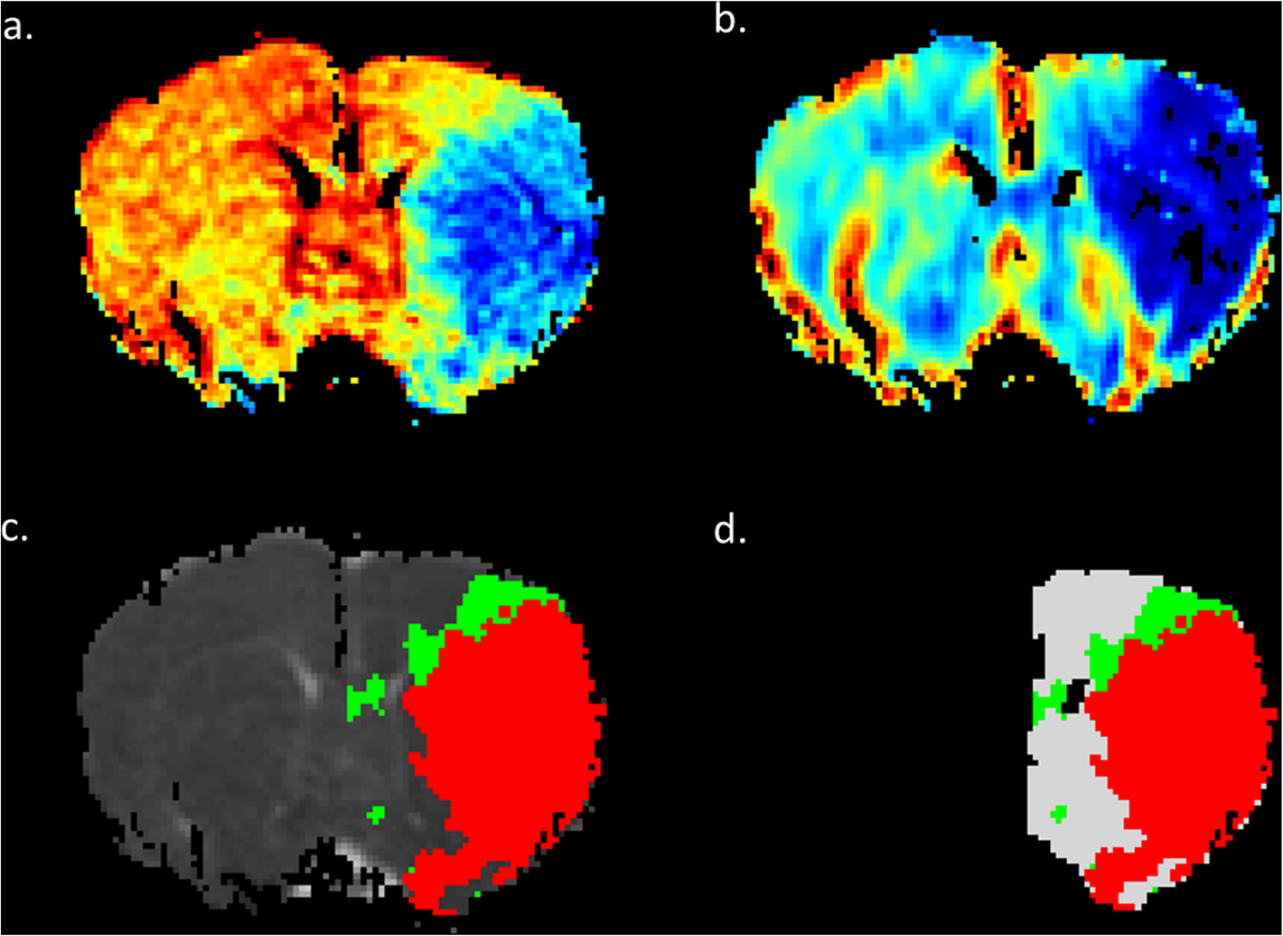
Definitions of the Ischemic Penumbra (IP), Infarct core (IC), and Normal Tissue (NT) in a Rat Subjected to Permanent Middle Cerebral Artery Occlusion (pMCAO). IC was defined as the blue area in the mean diffusivity (MD) map (**a**) and perfusion deficit at 0.5 h after pMCAO is shown in (**b**). Perfusion–diffusion mismatch is illustrated in (**c**) and (**d**), where the red region indicates the IC and the green region indicates the IP. The NT was defined as the region in the ipsilateral hemisphere except for the IP and IC [white in (**d**)]. **d** Indicates the “label” for the IC, IP, and NT

With supervised learning procedures in the classifiers, the labels of the IP, IC, and NT should be determined in advance. According to previous research, abnormal MD (i.e., IC) is defined using a reduction of 30% of the contralateral hemisphere with the exclusion of the ventricles (Fig. 1a) [17]. Perfusion deficit is defined with a lower CBF threshold of 46% reduction of the contralateral hemisphere (Fig. 1b) [18]. The rCBF map was coregistered to the MD maps to delineate the perfusion–diffusion mismatch (i.e., the IP). Regions without CBF deficit within the ipsilateral brain were defined as the NT. Contiguity correction was performed to remove “misclassified” pixels (Fig. 1c) [19, 20]. Finally, as shown in Fig. 1d, the regions of the IP, IC and NT were depicted and the corresponding voxels were labeled.

#### Feature extraction

Each voxel was characterized by 110 features and was segmented into the IP, IC, or NT according to the definitions in the previous section. Three types of features (the relative DTI-derived metrics; Mahalanobis distance; normalized histogram described by kurtosis, skewness, and bin counts) were extracted from regions of interest in the voxel-located slices and adjacent slices. These features represented the spatial patterns around the voxel as well as the spatial relationship between the voxel and its neighboring voxels.

#### Relative DTI-derived metrics

Six relative DTI-derived metrics (rMD, rAD, rRD, rFA, rL, and rq) were obtained for each pixel. Once the regions of the IP, IC and NT were depicted, the relative DTI metrics were derived on a pixel-by-pixel basis in relation to the contralateral homologous tissue as follows: r*X* = (*X*_ipsilateral_ − *X*_contralateral_) / *X*_contralateral_, where *X* indicates the particular DTI index. In consideration of the lesion spatial contiguity, metrics of two vertical adjacent voxels in the adjacent slices were also used. Thus, a total of 18 DTI-derived features were computed for each voxel.

#### Mahalanobis distance

**Fig. 2:**
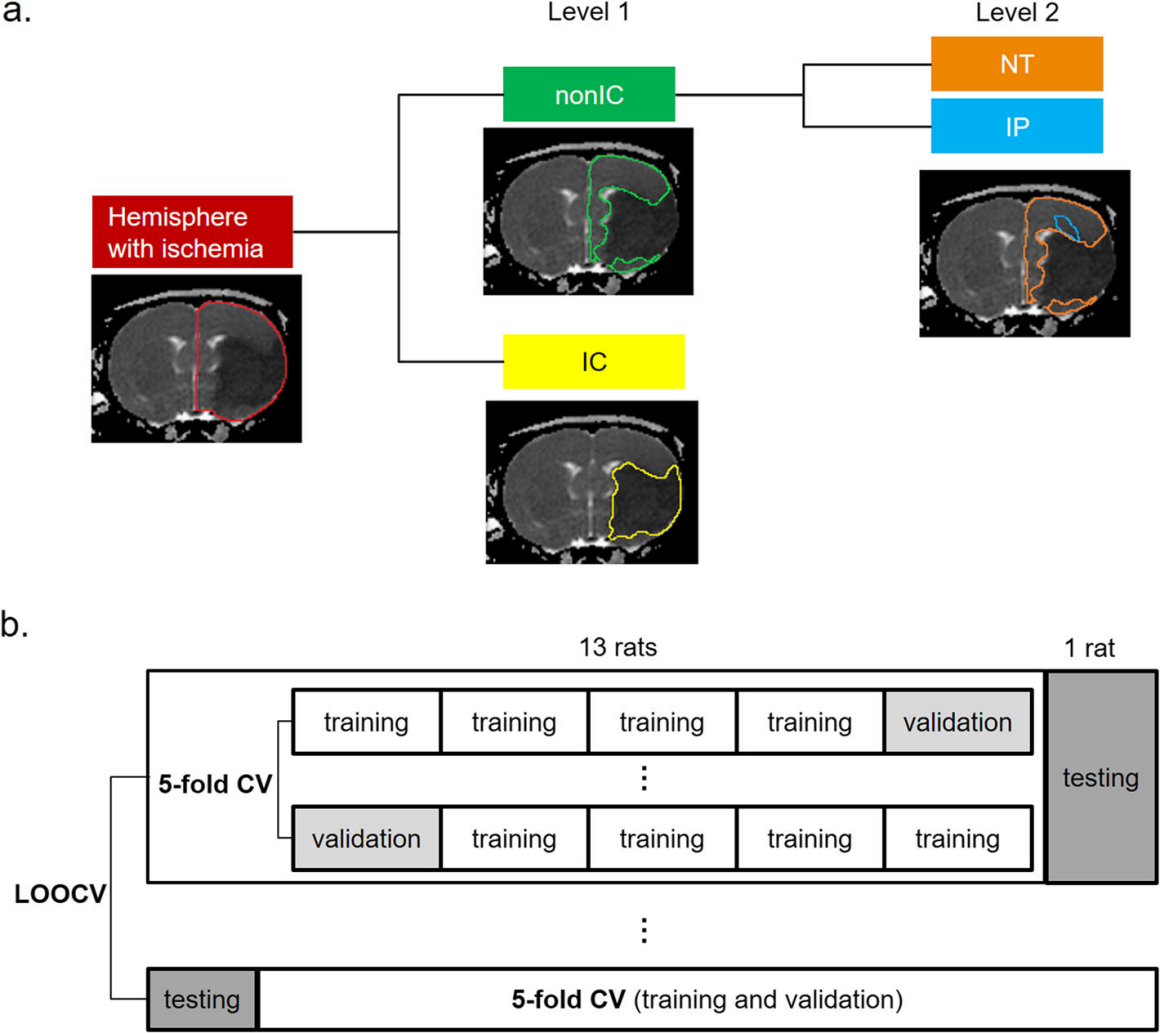
Strategy of 2-level Classification and Validation. **a** The proposed 2-level strategy for voxel-wise classification to classify every voxel in the hemisphere into a tissue subtype (i.e., the IC, IP, or NT). **b** The validation methods include 5-fold cross validation in training phase and leave-one-out cross validation for the final prediction

Although previous studies have adopted the spatial Euclidean distance from the lesion location as features [21, 22], various ischemic distributions and multifocal ischemia make stroke lesion segmentation difficult [21]. In this study, once the IC and non-IC were classified by the first-level classifier (Fig. 2a), the Mahalanobis distances [19] between each non-IC feature vector and the distribution of all IC feature vectors were calculated as discriminative features for the second-level classifier.

#### Normalized histogram (kurtosis, skewness, and bin counts)

The normalized histogram-specified 11 bins of the relative DTI metrics in an area of the 7 × 7 window in the coronal planes were calculated [21]. In addition, we reconstructed the axial projection maps from the coronal slices to calculate the normalized histogram in an area of the 3 × 3 window in the axial planes. The kurtosis, skewness, and bin counts of each histogram distribution were calculated as features. A total of 90 features were obtained for each voxel.

#### Two-level classification

We propose a 2-level classification model composed of two binary classifiers to hierarchically classify the stroke hemisphere into three tissue subtypes, as displayed in Fig. 2a, and the classification performance was evaluated through the leave-one-out cross validation (LOOCV) method (i.e., 13 rats were used for training and a remaining rat for testing each time, Fig. 2b). A total of 141,806 samples (voxels) were obtained from 14 rats within 71 slices. During the training phase, five-fold cross validation (CV) was used to prevent the possible bias of overfitting. We trained the first-level classifiers by using the 18 DTI features to classify hemisphere into the IC and non-IC. We then used all 110 features for the second-level classifiers. For comparison, a single-level classification model using either support vector machine (SVM), k-nearest neighbors (KNN), or decision tree algorithms was constructed to classify three tissue subtypes simultaneously based on 18 DTI features. The classification methods were implemented using the Statistics and Machine Learning Toolbox in the MATLAB.

#### Validation and statistical analysis

The performance of the classification was evaluated by calculating the accuracy, sensitivity, specificity, and areas under the receiver operating characteristic curve (AUC). The slice-to-slice correspondence analysis between the estimated lesion volume and the volume defined by PDM was also conducted. To evaluate the applicability and potential of our proposed approach for the whole ischemic lesion volume, the Mann–Whitney U-test was also used for comparison between the average estimated lesion volumes and those defined by PDM. Statistical tests were performed using SPSS® (Version 19.0; SPSS Inc., Chicago, IL, USA). All the group data are reported as mean ± SD, and the significance level was defined at a *p* value < .05.

## Results

**Fig. 3:**
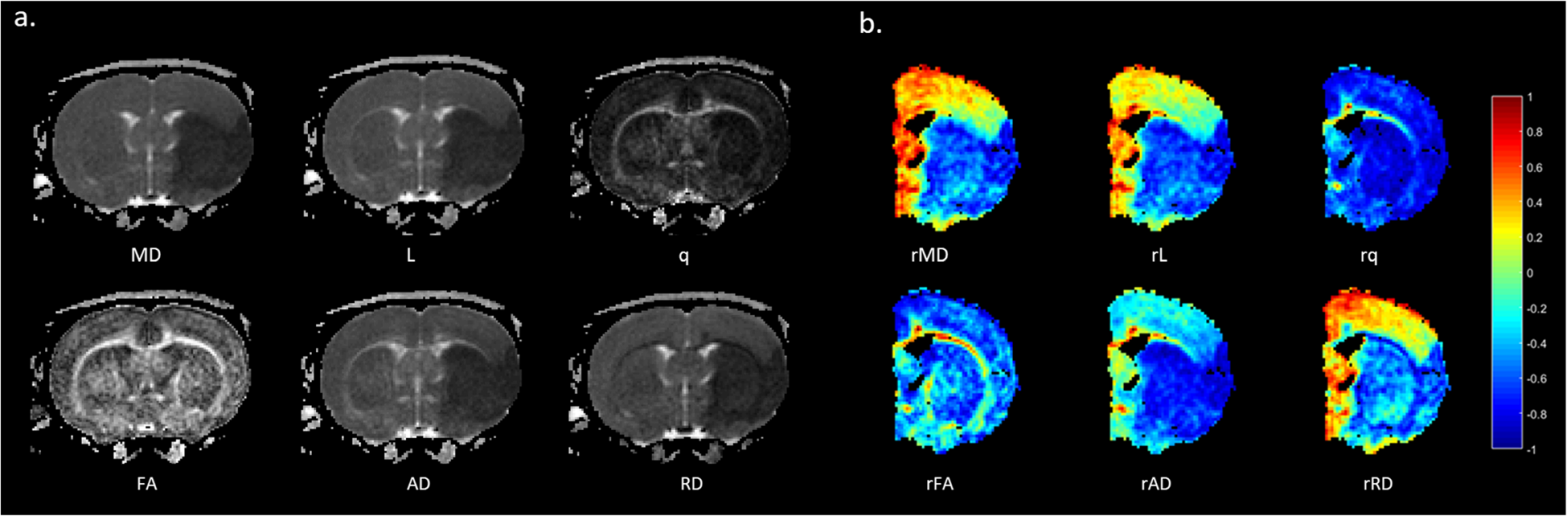
Maps of Diffusion Tensor Imaging (DTI) Metrics Measured at 0.5 Hours After pMCAO. **a** Significant hypointensities on the ischemic lesion can be observed from MD, L, q, AD and RD maps but not from FA map. **b** The relative DTI metrics are shown. The intensity of the map represents the quantitative decreases or increases of the DTI metrics compared with the corresponding contralateral homologous tissue

Figure 3a illustrates the maps of DTI metrics at 0.5 h post-pMCAO. The q, L, MD, AD, and RD maps demonstrate initial hypointensity changes in the ischemic areas while the FA map exhibits symmetrical signal intensity. Figure 3b shows six relative DTI-derived metrics (rMD, rAD, rRD, rFA, rL, and rq) obtained for each voxel.

For the 2-level classifiers, the SVM algorithm revealed the best overall discrimination performance. Its first-level classifier exhibited a high performance for discriminating the IC from the non-IC in the training dataset (AUC: 0.99; accuracy: 96.3%; sensitivity: 0.95; specificity: 0.97). The discrimination performance remained high for the second-level classifier (IP vs. NT), with AUCs, accuracies, and specificities up to 0.96, 95.0% and 0.97, respectively. However, the sensitivities were relatively low (0.85 ~ 0.86). The detailed classification performance using the LOOCV is listed in Table 1. On the other hand, the single-level classifiers exhibited poor sensitivities for IP detection (SVM: 29.0%; KNN: 46.8%; decision tree: 4.9%), as shown in Table 2.

**Table 1:** Performances of the 2-level classifiers for the training dataset

		Classifier performance		
		SVM	KNN	Decision tree
AUC	IC vs nonIC	0.99 ~ 1	0.99 ~ 1	0.99
	IP vs NT	0.96 ~ 0.98	0.98	0.78 ~ 0.80
Accuracy	IC vs nonIC	96.3 ~ 96.7%	96.4 ~ 96.6%	95.4 ~ 95.8%
	IP vs NT	95.0 ~ 95.9%	94.3 ~ 94.8%	84.3 ~ 85.5%
Sensitivity	IC vs nonIC (true rate for IC)	95 ~ 96%	95 ~ 96%	94 ~ 96%
	IP vs NT (true rate for IP)	85 ~ 86%	80 ~ 81%	30 ~ 36%
Specificity	IC vs nonIC (true rate for nonIC)	97 ~ 98%	97%	97%
	IP vs NT (true rate for NT)	97 ~ 98%	98%	95 ~ 97%

**Table 2:** Performances of the single-level classifiers for the training dataset

Classifier	Accuracy	Sensitivity for IC	Sensitivity for IP	Sensitivity for NT
SVM	81.7%	97.0%	29.0%	79.1%
KNN	90.5%	96.0%	46.8%	95.0%
Decision Tree	86.1%	95.4%	4.9%	95.6%

The trained SVM classifiers were then applied to the testing dataset, and the results are presented in Table 3. The classification accuracy of the IC and non-IC segmentations was 96.0 ± 2.3%. For further subclassification within the non-IC, the classification accuracy of IP and NT segmentation was 80.1 ± 8.0%. Overall, the classification accuracy of the segmentation of the 3 tissue subtypes was 88.1 ± 6.7%, and the median was 90.0% within the cerebral hemisphere of stroke (ranging from 69.5 to 96.9%). We observed a favorable classification outcome for the testing rats.

**Table 3:** Performances of the 2-level SVM Classifiers for the Testing Dataset

	Accuracy	Range	Median
IC vs. nonIC	96.0 ± 2.3%	88.2 ~ 98.9%	96.7%
IP vs. NT	80.1 ± 8.0%	61.0 ~ 92.6%	81.5%
hemisphere (IC + IP + NT) vs. PDM	88.1 ± 6.7%	69.5 ~ 96.9%	90.0%

**Fig. 4:**
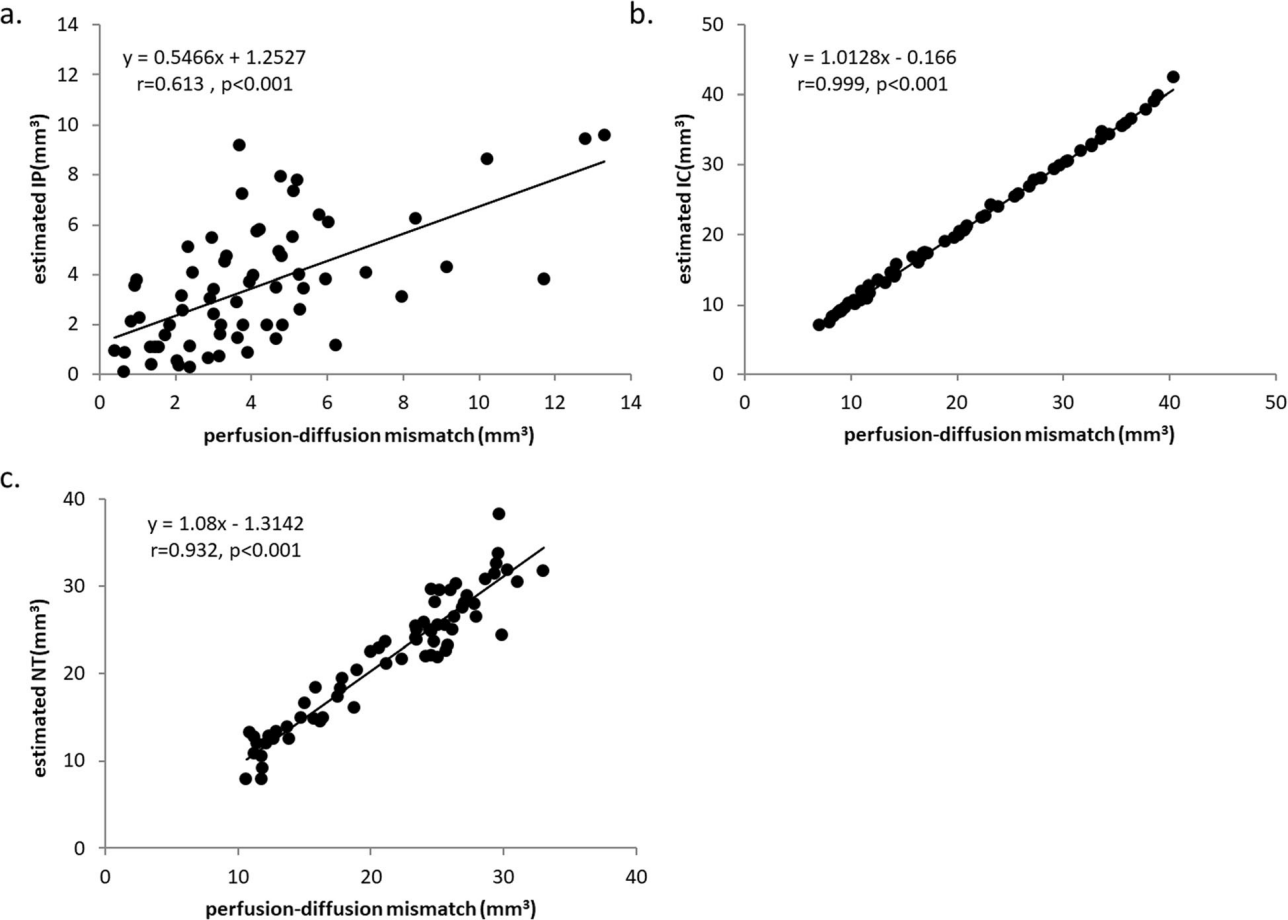
Results of Slice-to-Slice Correspondence Analysis Between the Classifier-Estimated Volume and the Perfusion-Diffusion Mismatch -Defined Volume for the IP (**a**), IC (**b**), and NT (**c**)

The slice-to-slice correspondence analysis between the classifier-estimated volume and the corresponding volume defined by PDM for the 3 tissue subtypes in all 71 slices is illustrated in Fig. 4. The correlation coefficients between the volume estimated from one slice and the volume defined by PDM were 0.613, 0.999, and 0.932 (with all *p* < .001) for the IP, IC, and NT, respectively. The estimated IC and NT volumes exhibited excellent correlations with the conventional PDM measures, whereas the IP exhibited a moderate correlation.

**Fig. 5:**
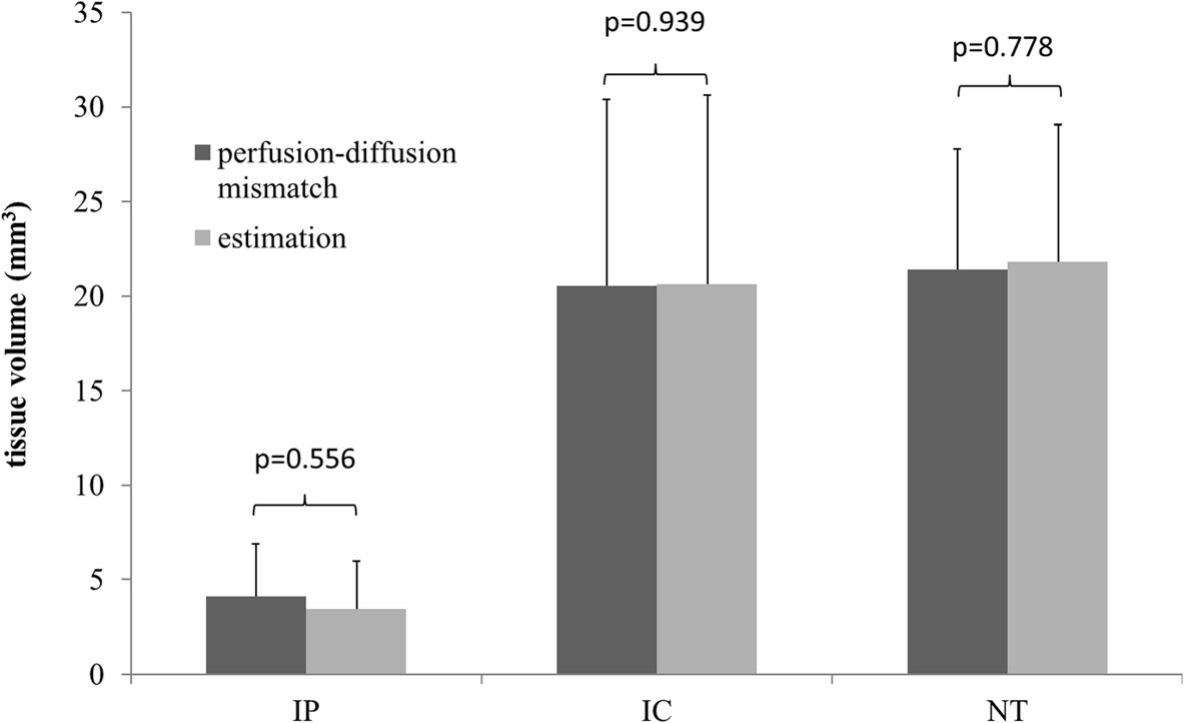
Average of the Estimated Volume and Perfusion-Diffusion Mismatch Defined Volume for the IP, IC, and NT for the 14 rats. No significant differences were observed between the classifier-estimated volume and the perfusion-diffusion mismatch defined volume in the IP (*P* = .56), IC (*P* = .94) and NT *(P* = .78)

Figure 5 depicts a comparison of the average of the classifier-estimated lesion volumes with the average of the volumes defined by PDM across 14 rats for the 3 tissue subtypes. The volumes of the 3 tissue subtypes were calculated and summed up across slices. Overall, the classifier-estimated lesion volume was comparable with those defined by PDM-defined lesion volume in all tissue subtypes. In the Mann–Whitney U-test, the estimated IP (17.8 ± 10.4 mm^3^), IC (104.8 ± 72.6 mm^3^), and NT (110.7 ± 55.9 mm^3^) volumes were not significantly different from those defined by PDM for the IP (20.9 ± 10.5 mm^3^, *p* = .56), IC (104.3 ± 73.2 mm^3^, *p* = .94), and NT (108.7 ± 56.8 mm^3^, *p* = .78) volumes.

**Fig. 6:**
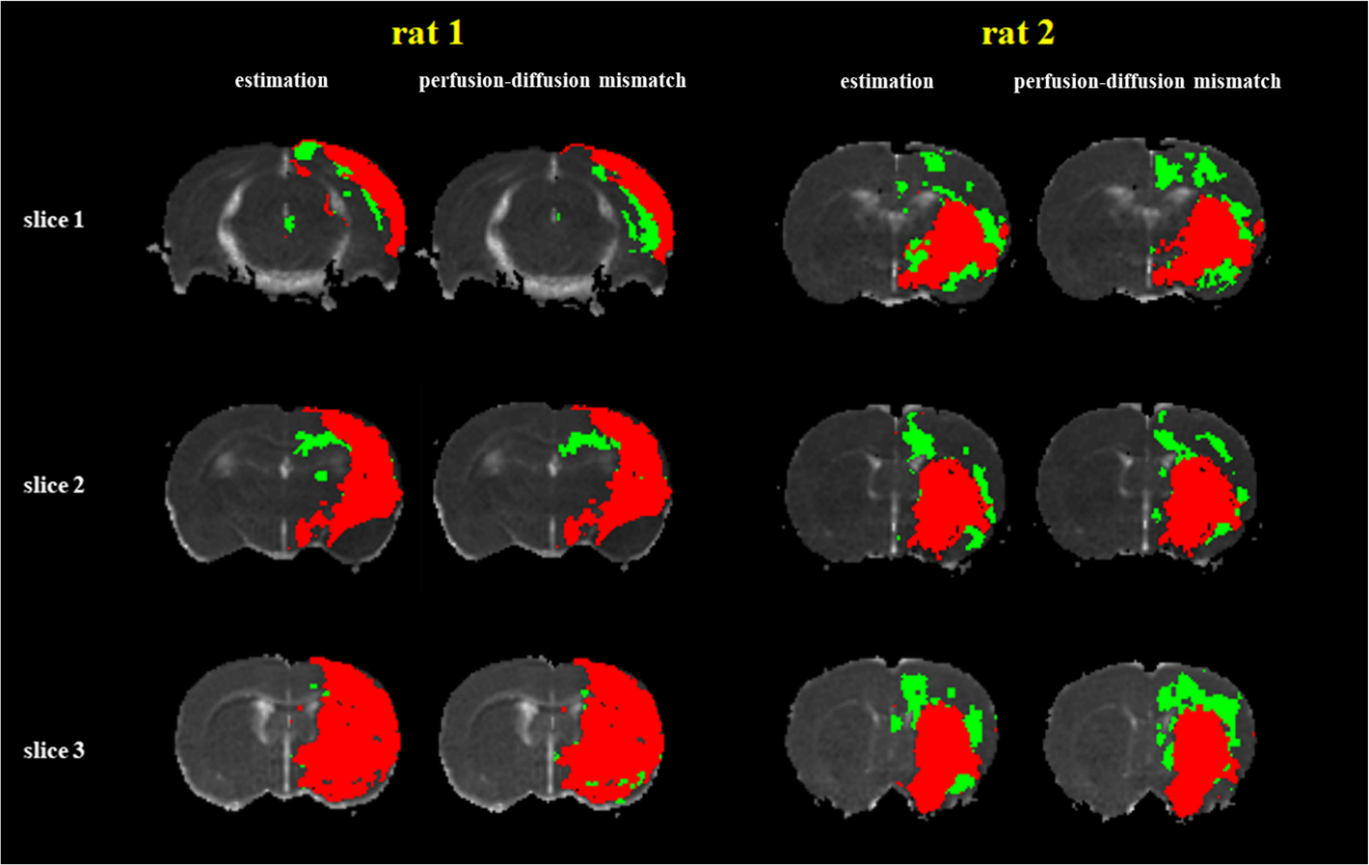
Demonstration of a Predicted Mismatch for 2 Rats. The conventional perfusion–diffusion mismatch and estimated mismatch are illustrated, where the red region indicates the IC and the green region indicates the IP. The NT is displayed in grayscale

Figure 6 illustrates the comparison of the classifier-estimated IC and IP with the corresponding PDM-defined IC and IP for a rat. In the suture-occlusion model, the IP is relatively small (even sparse) in areas at the margin of a large IC. Nevertheless, the proposed classification model can successfully segment the ischemic regions into the IP and IC through visual inspection.

## Discussion

In the present study, we developed a 2-level classification model with an overall accuracy of 88.1 ± 6.7% for discriminating the stroke hemisphere into the IC, IP, and NT regions on a voxel-wise basis in a pMCAO model. According to the analysis results, we suggest that a single DTI sequence combined with ML algorithms is capable of dichotomizing ischemic tissue into the IC and IP, which are comparable to the conventional PDM and DTI studies. This classifier system could assist clinical AIS management for acute triage and patient selections without the use of intravenous gadolinium-based contrast agent. Thus, the costs for contrast agent, the time for IV access, and the possibility of nephrogenic systemic fibrosis in renal-insufficient patients can be avoid [23]. Most previous imaging studies using ML tools have focused on diagnostic accuracy and prediction of prognosis after stroke [14, 24]. The current study moved a step further on the development of a quick and well-validated classifier to depict the salvageable tissue during an AIS phase.

Currently, the therapeutic time window for patients with AIS who are eligible for intravenous thrombolysis is within 4.5 h of onset [25–27]. In the DEFUSE 3 and DAWN trials, the time window for intra-arterial thrombectomy was extended up to 16–24 h [2, 5, 28]. These penumbra-based stroke trials have shown that patients with favorable clinical response generally have a relatively small IC (volume < 70 cm^3^) and large penumbra (PWI volume/DWI volume ratio > 1.8). Therefore, a quick and direct measurement of the IP at the acute phase through single imaging acquisition could be considerably helpful in stroke trials. Recent studies employing DTI metrics to characterize the IP after pMCAO have shown a persistent IP up to 6.5 h. DTI metrics exhibit promise in differentiating the IP from the oligemia and IC and even in determining the time of stroke onset [8]. DTI has been applied to measure the cerebral microstructural integrity of cell membranes after ischemia by characterizing their tensor magnitude, orientation, and anisotropy such that different tissue injuries can be stratified. The proposed 2-level binary classification is based on the same principle that DTI metrics could provide information about the varying degree of microstructural damages following ischemic changes. The combination of DTI metrics with ML algorithms could thus enable the precise stratification of tissue subtypes, which would lead to quick estimation of the salvageable tissue in the acute phase of a stroke.

In our study, the classifier exhibited a strong correlation (*r* = 0.999, *p* < .001; Fig. 4b) between the estimated IC volume and abnormal MD-defined IC volume. However, only a moderate correlation could be achieved between the estimated IP volume and the PDM-defined volume (*r* = 0.613, *p* < .001; Fig. 4a). Several factors may have caused the moderate correlation. One factor is that the intraluminal suture MCAO model may produce variable lesion distributions and the CBF reduction can be highly heterogeneous [29–31], which presumably leads to CBF-based viability threshold deviation in the range of ± 11% [18]. In other words, the deviation can result in overlapping measurements at the borders between the IP and NT areas, which leads to inappropriate preassigned class for ML. Another factor is the minor changes of DTI metrics in the IP (decrease approximately 10%) during the hyperacute stage. Such changes have been reported in humans [32, 33] and in rodent models [8, 11]. The small difference in DTI metrics between the IP and NT (an approximately 40% difference in DTI metrics between the IC and NT) that are challenging to separate in the original or even feature space may have contributed to the relatively low accuracy of discrimination between the IP and the NT (80.1 ± 8.0%; Table 3). Nonetheless, our findings showed that the classifiers can assist in estimating the IP and IC volumes, which are comparable with the lesion volumes calculated from conventional PDM (all *P* > .556, Fig. 5). The proposed classifiers may also be used for the 14–27% of patients who have unknown onset time (such as in wake-up strokes) [34] but still have substantial salvageable tissue volumes. Nonetheless, IP gradually diminish and eventually progress into IC tissue without recanalization. Previous study, however, has shown 100% normobaric hyperoxia (NBO) was helpful to “freeze” the IP and prolong the treatment time window [35]. Combined the proposed classifiers may help to select a candidate for reperfusion and evaluate the treatment efficacy repeatedly.

Limited studies have applied ML algorithms for the discrimination of the IC and salvageable tissue. A study using 2 convolutional neural networks demonstrated a 94% lesion detection rate by training the segmented IC in DWI from 741 patients [36]. Despite the difference in ML algorithms, the results of the aforementioned study were similar (approximately 95–96% for the IC; Table 1) to the results obtained with the support vector machine (SVM)-based model in this study. The high IC detection rates obtained are probably attributable to the inherent high sensitivity of DWI in depicting acute infarct [37–40]. Our classifier can achieve not only a high IC detection rate but can also separate the non-IC into the IP and NT through DTI alone. An animal study by Huang et al. [41] used an SVM-based model to predict acute ischemic tissue fate through the CBF and apparent diffusion coefficient mismatch. The AUCs in the 30-min, 60-min, and pMCAO groups were 88 ± 2 .9%, 94 ± 0.8%, and 97 ± 0.9%, respectively. The marginally lower performance of our 2-level classification model was because we did not use the perfusion parametric maps as features in our training process. Multimodal MR features from different imaging sequences, such as contrast enhancement, T2 fluid attenuation inversion recovery, and T1/T2-weighted imaging, have been shown to enhance the performance of ML-based approaches, either in lesion segmentation or the prediction of tissue outcome [21, 22, 41, 42]. However, adding multiple MR sequences may result in long scanning times, which may not be feasible for patients with AIS simply because “time is brain.”

The performance of classifiers highly depends on the features extracted from the images. Although the end-to-end deep learning model, which covers both feature extraction and classification procedures, has exhibited suitable capability for classifying natural images, the small samples size of medical images limits its reliability in clinical applications. Radiomics is a recently developed computational pipeline for extracting quantitative features from medical images [43, 44]. These features include first-order statistical features, shape-based features, and the most used texture features in oncology studies [43–45]. In the current experimental model, we used first-order statistical features (i.e., skewness and kurtosis), shape-based features (i.e., distance measures for similarity [21, 22]), and texture features for the spatial information of the lesions. Due to the relatively small number of rats and imbalanced distribution of the IC, IP, and NT, we adopted the LOOCV in the training phase to obtain a larger number of training data than that obtained using the *K*-fold partition scheme [46]. This approach also provides an unbiased evaluation of the performance [47]. The small number of rats made the usage of a convolutional neural network a challenge because the training procedure was based on the entire image. However, the pixel-wise analysis along with SVM provided sufficient samples (*N* = 141,806) for model training as well as a reasonable ratio of samples to data dimensions (*d* = 110). To avoid the possible problem of overfitting, we adopted a 5-fold CV for evaluating the proposed methods. In addition, estimation of volumetric IP and localization would be implemented by reconstruction of 3D from 2D results to precisely know how the spatially location relative to normal tissue is, which may help physicians’ assessment before reperfusion surgery.

This study has some limitations. First, correctly labeled data is essential in supervised learning such as SVM. Our research used a PDM overlay for DTI segmentation. The conventional PDM region may exceed the true penumbral area and is usually much larger than the final size of the infarct [48–50]. The perfusion deficit overestimates the region at risk, including the penumbral tissue and benign oligemia [51]. Other modalities, such as positron emission tomography [52, 53] or MRI using oxygen challenge with T2* signal change [54], could provide detailed insight into the complex pathophysiological changes of the brain after ischemia and may be used to additionally define the penumbral tissue. In our study, we adopted 46% reduction of CBF to define penumbra by referencing Meng’s result [18]. With this criterion, we also observed the consistency with the final infarct regions in the T2-weighted images. Second, possible redundant features may be eliminated through advanced feature selection or dimensionality reduction methods to improve the accuracy and reduce the computational time. Also, because features are essential in classification, further efforts should be made to explore stroke-related features. Finally, the region-wise histopathological correlation, which is difficult to perform because of the dynamic changes within the penumbra, may be implemented to provide further proof of the value of our proposed classifiers.

## Conclusions

Our results suggested that single DTI combined with ML algorithms could provide a noninvasive, quick, and reliable method of assessing the salvageable tissue, thus accelerating the management of patients with AIS. To the best of our knowledge, this is the first ML-based study to demonstrate the potential of using a single DTI sequence for substituting the conventional approach of PDM, offering a practical workflow for clinical decision-making and stroke trials.

## Data Availability

The data generated or analyzed during the current study is not publicly available due to restrictions in the ethical permit, but may be available from the corresponding author on request.
